# Magnesium and Migraine

**DOI:** 10.3390/nu17040725

**Published:** 2025-02-18

**Authors:** Ligia J. Dominguez, Nicola Veronese, Shaun Sabico, Nasser M. Al-Daghri, Mario Barbagallo

**Affiliations:** 1 Department of Medicine, “Kore” University of Enna, 94100 Enna, Italy; 2 Geriatric Unit, Department of Medicine, University of Palermo, 90127 Palermo, Italy; nicola.veronese@unipa.it (N.V.); mario.barbagallo@unipa.it (M.B.); 3 Chair for Biomarkers of Chronic Diseases, Biochemistry Department, College of Science, King Saud University, Riyadh 11451, Saudi Arabia; ssabico@ksu.edu.sa (S.S.); ndaghri@ksu.edu.sa (N.M.A.-D.)

**Keywords:** magnesium, migraine, headache, pain

## Abstract

Migraine is a widespread and intricate neurological condition that involves various factors and is marked by recurring headache episodes. Migraine is among the ten neurological conditions accounting for the greatest disability in the whole population, the leading cause of disability for children and adolescents aged 5–19 years, and the second cause of disability for adults aged 20–59 years. Magnesium deficiency is also a very common condition resulting from diverse reasons, including insufficient dietary consumption or increased loss through the gastrointestinal or renal system. Accumulated evidence from case reports, case–control studies, observational studies, and randomized, placebo-controlled trials has shown the effectiveness of magnesium supplementation in alleviating migraine, both acutely and chronically. Mechanisms that may help explain these results include the potential link between magnesium deficit and spreading cortical depression, vascular changes, oxidative stress, chronic inflammation, nervous excitation, neurotransmitter release, and electrolyte imbalances. This article aims to provide a comprehensive review of the available evidence on the links between magnesium and migraine, considering the role of magnesium in the pathogenesis of migraine and the utility of magnesium in its prevention and treatment.

## 1. Introduction

Migraine is a common and complex neurological disorder that involves multiple factors and is characterized by recurrent headache episodes [1,2,3]. It can manifest in either episodic or chronic forms, with or without aura, and often accompanied by various transient motor and sensory disturbances. Historically, migraines were referred to as “hypoglycemic headaches” in the early 20th century [1]. Migraine is typically defined by moderate to severe, throbbing, unilateral headaches associated with symptoms such as nausea, photophobia, phonophobia, and vomiting [2,3]. The term “migraine” comes from the Greek word “hemicranias”, meaning “half of the head”, which reflects the common experience of pain on one side of the head, although bilateral pain, affecting both the front and the back of the head, is also common. The pain is usually throbbing and intensifies with physical activity or movement. Research into the mechanisms behind migraines has highlighted the activation of the trigeminovascular system as a key factor, with both genetic and environmental influences playing significant roles in their development [1,2,4]. The pathogenesis of migraine has also been linked to energy deficit syndrome [5].

Migraine is a relevant disabling condition, affecting approximately 15% of the general population annually [2,6]. According to the latest 2024 report on the global, regional, and national burden of disorders affecting the nervous system, 1990–2021 [6], migraine is among the ten conditions that accounted for the greatest nervous system disability-adjusted life years (DALYs) in the whole population in 2021 together with stroke, neonatal encephalopathy, and Alzheimer’s disease, among others. For older children and adolescents aged 5–19 years, migraine was the leading cause of DALYs followed by neurological complications due to preterm birth, and epilepsy. For adults aged 20–59 years, migraine was the second cause of DALYs after stroke, followed by diabetic neuropathy [6].

Although the pathophysiology of migraine is still not completely clear, in recent years there have been important advances in knowledge about mechanisms that help explain it and new therapeutic targets that have led to the availability of new pharmacological treatments [7]. However, there are also dietary components and other environmental determinants that seem to contribute to the genesis of migraine, including magnesium insufficiency.

Magnesium is a crucial mineral that plays an essential role in numerous cellular functions, including over 600 enzymatic reactions, energy production, oxidative phosphorylation, protein synthesis, nucleic acid synthesis and stability, and carbohydrate metabolism [8,9,10,11]. Maintaining adequate magnesium levels is vital for the proper functioning of organs and systems in the human body, including the nervous system. Magnesium deficiency is prevalent in older adults and has been linked to several age-related chronic conditions and other diseases such as migraine [11,12,13].

Accumulating research indicates a link between magnesium deficiency and acute and chronic migraines, ranging from case reports to case–control studies, observational studies, and randomized controlled trials (RCTs), which will be reviewed in the present article. Magnesium offers an alternative to or an opportunity for combination therapy with conventional medications, which may pose concerns such as high costs and side effects. Magnesium has been used for numerous health conditions including migraine [11,12,13], demonstrating safety and minimal side effects, which renders it a particularly appealing possibility for use in populations where side effects are less tolerable, including children, pregnant women, and older adults.

The present article aims to provide a comprehensive review of the available evidence of the links between magnesium and migraine, considering the role of magnesium in the pathogenesis of migraine and the utility of magnesium in its prevention and treatment.

## 2. Classification, Epidemiology, and Pathophysiology of Migraine

### 2.1. Classification

According to the 2018 International Classification of Headache Disorders, 3rd edition [3], migraine is classified into two main types: (i) migraine without aura, a clinical condition characterized by headaches with distinct features and related symptoms; (ii) migraine with aura, mainly marked by temporary focal neurological symptoms that typically occur before or sometimes during the headache. Some individuals may also experience a prodromal phase, which occurs hours or days before the headache, and/or a postdromal phase after the headache subsides. Symptoms during the prodromal and postdromal phases can include hyperactivity, hypoactivity, depression, specific food cravings, frequent yawning, fatigue, and neck stiffness or pain. In addition, chronic migraine is characterized by headaches occurring on fifteen or more days per month for a duration of over three months. On at least eight of these days each month, the headaches exhibit the typical features of a migraine [3].

### 2.2. Epidemiology

The recently published systematic analysis of the global, regional, and national burden of disorders affecting the nervous system estimated mortality, prevalence, years lived with disability (YLD), years of life lost (YLL), and DALYs, with corresponding 95% uncertainty intervals (UIs), by age and sex in 204 countries and territories, from 1990 to 2021 [6]. Migraine was the third condition with the highest age-standardized DALYs in 2021, considering the whole population, after stroke and neonatal encephalopathy. The age-standardized prevalence of migraine globally in 2021 was 14,246.5 per 100,000 people, with a 1.6% increase with respect to 1990. Migraine age-standardized prevalence was higher among women (17,902.6 per 100,000) than among men (10,624.2 per 100,000). The ratio of women-to-men prevalence of migraine in 2021 was 1.62 (UIs 1.39–1.79), which was the third highest women-to-men ratio among those associated with disorders affecting the nervous system [6]. For older children and adolescents aged 5–19 years, migraine is the leading cause of DALYs (380.0 per 100,000 people), followed by neurological complications due to preterm birth (234.3 per 100,000 people) and epilepsy (185.1 per 100,000 people). For adults aged 20–59 years, migraine is the second cause of DAILYs (750.8 per 100,000 people), solely preceded by stroke (1126.1 per 100,000 people) [6].

### 2.3. Pathophysiology

To discuss about migraine pathophysiology we can briefly recall the history of the disease [14]. Migraine symptoms have been recognized since ancient times, initially attributed to supernatural forces and later to an imbalance of bodily humors. The Greek physician Aretaeus distinguished between cephalea and heterocrania (later renamed hemicrania by Galen). In the 17th century, Willis suggested that intracranial blood vessel dilation was linked to migraine, while in the 1860s Latham proposed vasomotor theories, including angioparalysis following sympathicotonia. The introduction of ergot and ergotamine supported the vasodilation theory. The discovery of serotonin and its agonists furthered migraine research, while in the 1940s, studies on aura and cortical spreading depression (CSD) linked these symptoms to blood flow changes. The identification of calcitonin gene-related peptide (CGRP) led to new pathophysiological explanations and to the development of new treatments [14].

Thus, even if chronic migraine was first recognized in the early 17th century [14], the underlying causes remained unclear throughout much of the 20th century. Initially, the understanding of migraine’s pathophysiology was primarily based on neurological or vascular mechanisms, with metabolic aspects being acknowledged only more recently [15].

**Figure 1:**
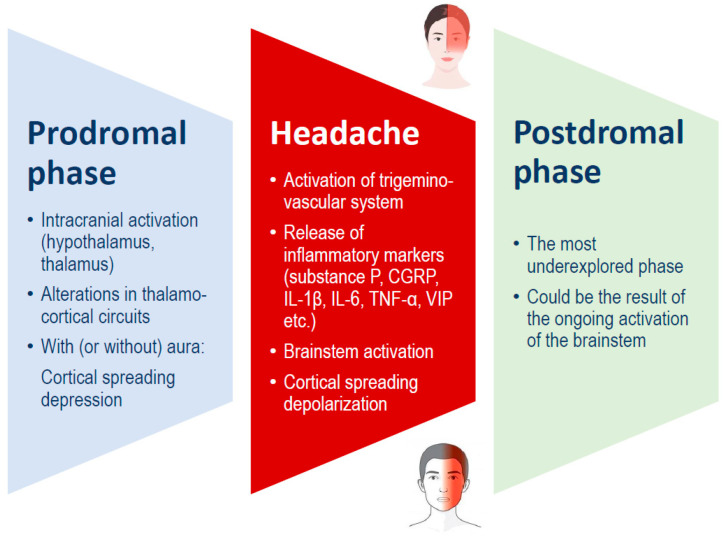
Migraine phases, which can overlap, and possible underlying mechanisms. CGRP: calcitonin gene-related peptide; IL: interleukin; TNF: tumor necrosis factor; VIP: vasoactive intestinal peptide.

Traditionally, a migraine attack consists of three stages: the premonitory phase, the migraine headache itself, and the postdrome phase (Figure 1). These phases may occur in sequence or overlap significantly. The premonitory phase usually begins 24 to 48 h before the headache and is commonly marked by symptoms such as fatigue, mood changes, and neck pain. Additionally, about one-third of the persons with migraines experience an aura, which includes temporary focal neurological symptoms such as visual, sensory, or motor disturbances. These symptoms may occur during the premonitory phase or simultaneously with the migraine headache phase [1,16].

Headache results from meningeal vasodilation and inflammation triggered by the activation of vascular networks [2,4]. The pathophysiology of migraine involves the modulation of pain originating from disrupted neural networks in the head [1,15]. Research has shown that the brainstem and diencephalic nuclei regulate the trigeminovascular system, which consists of efferent neurons that supply vascular networks and afferent neurons that transmit information to the trigeminal nucleus caudalis. Head pain is interpreted as meningeal inflammation and vasodilation due to the activation of these networks [4]. Neurotransmitters like serotonin play a vital role in both the pathophysiology and the treatment of migraine. Serotonin triggers an intracellular cascade that mediates inhibitory or excitatory neurotransmission. Serotonin receptors are widespread in the brain, including in pain-signaling circuits and cranial blood vessels [4,15].

## 3. Magnesium Role and Causes and Consequences of Its Deficit

### 3.1. Role of Magnesium in Cellular Functions

**Figure 2:**
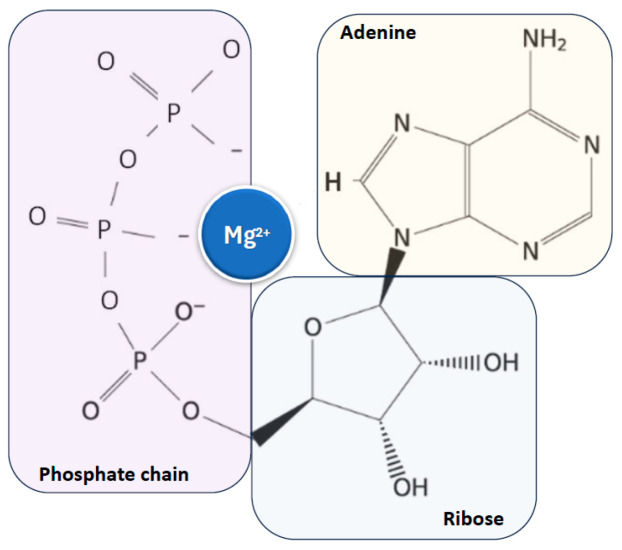
Close relationship between magnesium ion and ATP, the main cellular energy source. ATP: adenosine triphosphate.

Magnesium is found in all cells across organisms, from plants to higher mammals, and is essential for life and health due to its critical role as a cofactor for adenosine triphosphate (ATP), the main cellular energy source, to which it is closely linked [10,11,12,17,18] (Figure 2).

**Figure 3:**
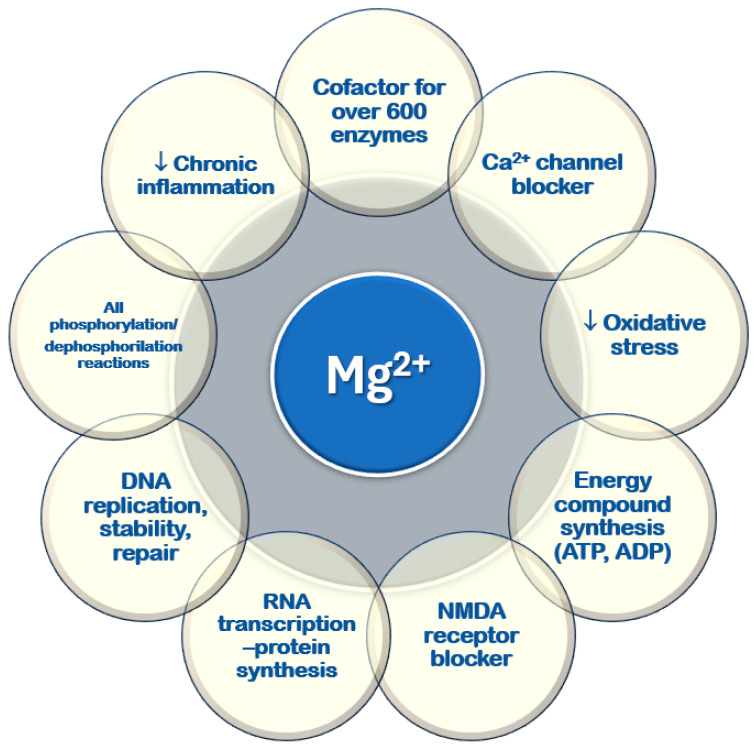
Cellular functions of magnesium. ADP: adenosine diphosphate; ATP: adenosine triphosphate; DNA: deoxyribonucleic acid; NMDA: N-methyl-D-aspartate; RNA: ribonucleic acid; ↓: reduced.

It plays a key role in various cellular and physiological processes, mainly through its ability to bind to nucleotides and regulate enzymatic activity [10,11,13,18] (Figure 3). All ATPase reactions require Mg^2+^–ATP, including those necessary for RNA transcription and DNA functions, replication, stability, and repair. Magnesium serves as a cofactor in over six hundred enzymatic reactions within every cell type. Additionally, it regulates glucose, lipid, and protein metabolism [19,20,21]. Magnesium is involved in neuromuscular function, vascular tone modulation, cardiac rhythm regulation, hormone secretion, and N-methyl-D-aspartate (NMDA) release in the central nervous system [22,23,24]. It acts as a second messenger in intracellular signaling [22,25,26,27] and helps regulate circadian clock genes that control biological rhythms [28].

### 3.2. Magnesium Defficiency

Chronic deficiency of magnesium is common, in particular among older adults [10,11,12]. However, insufficient magnesium intake extends to all ages, with estimates indicating that half of the US population has an inadequate dietary magnesium [29,30]. Magnesium deficiency can be attributed to several factors, such as a low dietary magnesium intake (typical in Western diets), increased urinary excretion, increased loss through the gastrointestinal tract, or reduced intestinal absorption due to various pathological and iatrogenic conditions [11,12,31]. The normal serum magnesium concentration in adults is 1.7 to 2.4 mg/dL (0.7 to 1.0 mmol/L). Hypomagnesemia is defined as serum magnesium levels below 1.7 mg/dL [12,32]. When severe hypomagnesemia is diagnosed, the underlying cause is typically evident from the patient’s history. However, if the cause is unclear, it is crucial to differentiate between renal and gastrointestinal magnesium losses using targeted diagnostic methods, such as the 24 h magnesium excretion and fractional excretion of magnesium tests and the magnesium loading test (MLT) [33,34,35,36]. The MLT was first used to confirm magnesium depletion in two healthy volunteers on a magnesium-deficient diet (1.1 mEq/day for 3–4 weeks), reporting a retention of 25% and 45% of the 8 h intravenously administered magnesium [37]. The MLT was later applied clinically in patients with gastrointestinal magnesium loss, where a retention of 20% or more was considered indicative of deficiency [38]. The same test with 1 h magnesium infusion was validated in healthy volunteers and renal transplant patients with magnesium wasting [35]. The MLT procedure involves an intravenous infusion of a magnesium salt (e.g., 0.1 mmol/kg of magnesium aspartate hydrochloride) in 500 mL isotonic saline over 1 h after emptying the bladder and monitoring vital signs. Blood samples for peak serum magnesium are drawn 60 min post-infusion, and urine is collected for 24 h. Magnesium retention is calculated as 1 − [Mg in 24 h urine/Mg test dose] × 100 [35]. Even if the MLT is considered a gold standard and has been used in diverse conditions, a standardized protocol is needed for consistent result comparisons across clinical studies [36].

**Figure 4:**
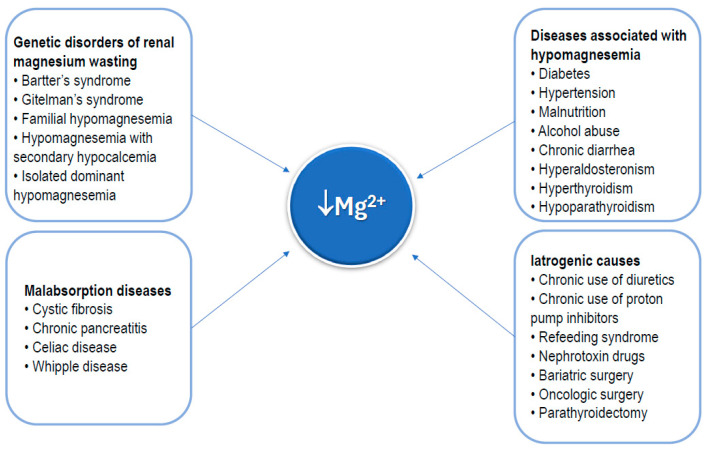
Main clinical conditions that can result in magnesium deficiency.

The main causes of hypomagnesemia are outlined in Figure 4. As shown, some rare genetic disorders may cause a chronic deficiency of magnesium [39]. Conversely, numerous common conditions can be associated with hypomagnesemia, including diabetes, hypertension, malnutrition (insufficient nutrient intake), alcohol abuse, chronic diarrhea, hyperaldosteronism, hypoparathyroidism, and hyperthyroidism [11,12,13]. Diseases causing global malabsorption, including celiac disease, cystic fibrosis, and acute and chronic pancreatitis, are also a cause of hypomagnesemia [40,41,42,43]. Commonly chronically used medications, such as diuretics and proton pump inhibitors [44,45], as well as nephrotoxin drugs [45], which promote loss of magnesium, should be considered as possible causes of hypomagnesemia. Radical surgical procedures of the gastrointestinal tract, such as bariatric surgery or extensive oncologic surgery for malignant gastrointestinal tumors, as well as parathyroidectomy, are also included in the list of causes of hypomagnesemia [46,47].

Additionally, reduced intracellular magnesium levels have been observed in older adults, even when the total serum magnesium level remains normal [48]. This phenomenon has also been noted in younger adults [49,50]. Mild magnesium deficiencies are often asymptomatic, and when symptoms do appear, they are usually non-specific and can be mistaken for common signs of aging or attributable to other disturbances and can be easily overlooked [11,12]. Several types of cardiac arrhythmias can reflect a state of hypomagnesemia, as well as electrolyte disturbances, in particular, hypokalemia, and hypocalcemia [51,52,53]. Of particular interest are the neuromuscular and central nervous system symptoms that include muscle cramps, weakness, fasciculations, tremors, lethargy, tetany, vertigo, nystagmus, and, in severe hypomagnesemia, convulsions and chorea-like movements [54,55].

**Figure 5:**
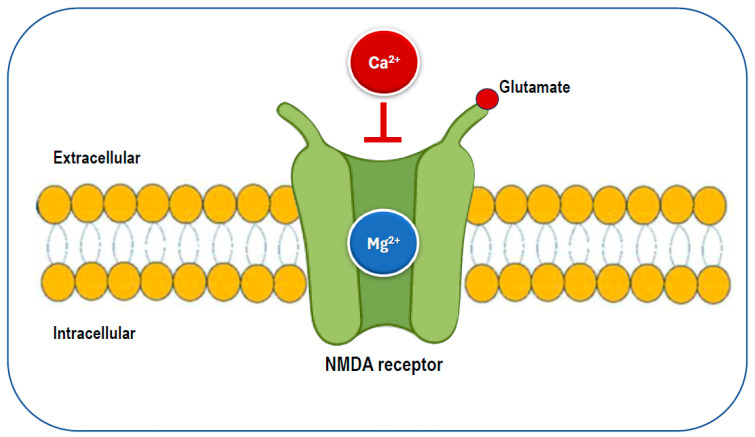
The glutamatergic N-methyl-D-aspartate (NMDA) receptor is blocked by magnesium, preventing the influx of calcium.

Given magnesium’s numerous functions, insufficient magnesium intake and levels may increase the risk of various health problems, including those associated with neurological conditions. Magnesium plays a key role in neurological function through its interaction with the NMDA receptor. It acts as a blocker of the calcium channel in the NMDA receptor (Figure 5), and its removal is necessary for glutamatergic excitatory signaling to take place [22,56]. When the magnesium levels are low, glutamatergic neurotransmission can be enhanced, creating a condition that favors excitotoxicity, which can contribute to oxidative stress and neuronal cell death [54]. Abnormalities in glutamatergic neurotransmission are associated with various neurological and psychiatric disorders [57,58], including migraine, chronic pain, epilepsy, Alzheimer’s disease, Parkinson’s disease, stroke, as well as depression and anxiety, which often co-occur with these conditions. Preclinical and clinical studies have shown that magnesium may have neuroprotective effects [59,60]. Therefore, magnesium may be a crucial dietary factor for preventing and/or treating these conditions.

In terms of magnesium intake, it has been shown that people with migraines tend to have diets of lower nutritional quality compared to those without migraines [61]. Furthermore, women with chronic migraines have been found to consume diets with lower nutritional quality than those with episodic migraines [62], which could lead to a reduced intake of magnesium overall. Slavin et al. performed a cross-sectional study on 3626 participants from the National Health and Nutrition Examination Survey between 2001 and 2004, quantifying dietary magnesium consumption in adults with migraine or severe headache in the US. The mean dietary consumption of magnesium was below the recommended dietary allowance (RDA) for both migraine and control groups. Achievement of the RDA through a combination of diet and supplements was associated with lower adjusted odds of migraine [63]. In fact, certain dietary factors, such as caffeine and alcohol, can trigger migraine, while others like magnesium and riboflavin may help alleviate migraine symptoms [64]. One way to enhance migraine management is through dietary changes that increase the intake of protective key nutrients. The Mediterranean diet, which emphasizes legumes, fish, whole grains, olive oil, vegetables, fruits, and nuts, is rich in both B vitamins and magnesium. Research has shown that following this diet is associated with a significant reduction in the frequency, duration, and severity of migraines [65,66]. Nevertheless, there is still no evidence that diet alone can treat migraine, but recommending a high-quality diet such as the Mediterranean diet rich in magnesium and other healthy nutrients is desirable for these patients.

## 4. Evidence for the Effects of Magnesium Supplementation on Migraine

### 4.1. Search Strategy

For the present article aiming to review the available evidence of the effects of magnesium supplementation on migraine in systematic reviews and clinical trials, we performed a search of the literature published in PubMed, from database inception to 1 December 2024, utilizing the following terms: “migraine” or “migraine disorders” or “episodic migraine” or “chronic migraine” and “magnesium” or “magnesium supplement” and “systematic review” or “meta-analysis” or “umbrella” or “randomized controlled trial” or “RCT” or “trial”. We enhanced the search by incorporating studies known to the authors and conducting additional forward citation searches. Studies focusing on the effects of supplements containing combinations of components including magnesium on migraines and dietary magnesium association with migraine were excluded. We also excluded other types of articles, such as editorials, comments, letters to the editor, case reports, case series, short communications, short reports, and perspectives. The focus was placed on the largest studies and the most recent publications.

### 4.2. Magnesium Levels in Migraine

Several studies have indicated a link between magnesium deficiency and headaches. Lower concentrations of magnesium have been reported in migraine patients in the serum [67,68] and saliva [68], intracellularly in erythrocytes and lymphocytes [69], and in both ictal and interictal brain regions in MR spectroscopy assessments [70]. Similar findings have been reported in other case–control studies [71,72,73]. A study utilizing a magnesium load test revealed increased magnesium retention in migraine sufferers compared to healthy controls, suggesting a systemic magnesium deficiency linked to migraine [71]. Additionally, a two-week trial showed that when migraine patients consumed mineral water with 110 mg/L of magnesium daily (ranging from 7.4 to 12 mmol per day for 15–18 days), their intracellular erythrocyte magnesium concentrations significantly increased, compared to those in healthy controls [69].

A study found that low serum magnesium levels caused a 35-fold increase in the likelihood of acute headaches in migraine patients [73]. This suggests that magnesium deficiency is an independent risk factor for the onset of migraines. Similarly, a study examined the relationship between clinical responses to 1 g of intravenous magnesium sulfate infusion for acute migraine and baseline serum ionized magnesium levels. The authors found a 50% pain reduction following the infusion in 87.5% of all participants with acute migraine, regardless of their serum magnesium level; however, in 86% of the patients with magnesium levels below 0.54 mmol/L, the relief lasted at least 24 h compared to 16% of those with serum ionized magnesium levels above 0.54 mmol/L [74]. Together, these findings support a link between magnesium deficiency and migraine and suggest magnesium deficiency as an independent risk factor for migraine occurrence. However, other research studies have found normal magnesium levels in the blood serum of migraine sufferers [75,76]. Interestingly, a study found that while the magnesium levels were within the normal range in patients with migraine and age-matched healthy controls, electrophysiological tests revealed abnormalities typically associated with hypomagnesemia [75]. The authors proposed a potential link between the frequency and severity of migraines and disturbances in neuromuscular excitability that depend on intracellular magnesium levels rather than on serum magnesium concentrations. Indeed, magnesium is a predominantly intracellular ion, while blood serum only contains about 1–2% of the total body magnesium [11]. The intracellular magnesium levels do not necessarily correlate with the overall serum magnesium levels: low intracellular magnesium has been found even in the absence of altered total serum magnesium [19,48,49,50]. A study found that in migraine patients with low intracellular erythrocyte and lymphocyte magnesium concentrations, the serum levels were normal [69]. In another study, only the magnesium loading test revealed a difference in the magnesium status in migraine patients compared to controls, whereas the serum levels were similar [71]. All this supports the notion that in migraine patients with normal serum magnesium levels there may be a latent magnesium deficiency contributing to the predisposition to migraine attacks.

### 4.3. Systematic Reviews and Meta-Analyses

Since the late 1980s, oral and intravenous magnesium have been considered potential treatments for migraine, with several meta-analyses and reviews evaluating their effectiveness over the past three decades. Table 1 shows the summary of the characteristics and results of systematic reviews and meta-analyses assessing the effects of magnesium supplementation on acute and chronic migraine. We found four systematic reviews [77,78,79,80], two systematic reviews and meta-analysis [81,82], one meta-analysis of RCTs [83], and one umbrella review [13], most reporting some significantly positive results (studies indicated in light blue in Table 1). Three out of the eight articles reported no clear benefit of magnesium supplements in patients with migraine [77,80,81], with two of them [77,81] concluding that there were not enough data to allow for stating with certainty the eventual benefit but not excluding that some benefit had been reported.

**Table 1:** Summary of the characteristics and results of systematic reviews and meta-analyses assessing the effects of magnesium supplementation on acute and chronic migraine *.

Authors Country Year	Type of Review	No. and Type of Studies Included	No. of Participants	Age Range (Years)	Intervention	Summary of Results
Teigen et al., USA, 2015 [77]	SR	4 RTCs	210	18 to 65	Oral Mg in migraine prophylaxis	The strength of the evidence supporting oral Mg supplementation is limited at this time.
Chiu et al., Taiwan, 2016 [83]	MA of RCTs	21 RCTs: 11 on i.v. Mg in acute migraine; 10 on oral Mg in migraine prophylaxis	acute migraine: 948 migraine prophylaxis: 789	28 to 46	i.v. Mg in acute migraine and oral Mg in chronic migraine	Significant acute migraine relief within 15–45 min, 120 min, and 24 h after i.v. Mg infusion (ORs = 0.23, 0.20, and 0.25, respectively). Oral Mg significantly alleviated the frequency and intensity of migraine (ORs = 0.20 and 0.27). Conclusions: i.v. and oral Mg should be adapted as parts of a multimodal approach to reduce migraine.
von Luckner et al., Switzerland, Austria, 2018 [78]	SR	5 RCTs	171	20 to 65	Oral Mg in migraine prophylaxis	One out of two Class I evidence trials showed significant reduction in migraine attacks vs. placebo; two out of three Class III trials evinced a significant reduction in the primary efficacy parameters vs. placebo. Conclusion: Grade C (possibly effective) evidence for prevention of migraine with Mg. Prophylactic migraine treatment with Mg dicitrate (600 mg) seems to be safe and cost-efficient in clinical use.
Miller et al., USA, 2019 [79]	SR	6 RCTs	503	>18	i.v. MgSO_4_ in acute migraine	Improved pain intensity with MgSO_4_ vs. comparators at 60–120 min, but not at 30 min. Endpoint results (>50% pain reduction) were conflicting (3 studies reporting improvement, no change, and less with MgSO_4_). Complete pain relief achieved with MgSO_4_ in 1 study and in aura subgroup in another. The need for rescue analgesia at any point was improved with MgSO_4_ in 1 study and in the migraine with aura subgroup in another; 24 h recurrence was improved with MgSO_4_ in 1 study, but unchanged in others. MA was not possible due to study heterogeneity. Conclusion: non-firm conclusion, but the existing evidence indicates potential benefits in pain control beyond 1 h, aura duration, and need for rescue analgesia.
Okoli et al., Canada, 2019 [81]	SR and MA	6 RCTs	542	3 to 65	Oral Mg in migraine prophylaxis	Based on insufficient evidence, it is unknown if Mg is effective for migraine prophylaxis in adults. High-quality, adequately powered RCTs are needed to fully evaluate the efficacy and safety of vitamins and minerals for migraine prophylaxis.
Park et al., France, Canada, Denmark, 2020 [80]	SR	3 RCTs	190	18 to 65	Oral Mg in migraine prophylaxis	One study found some benefit, where Mg demonstrated a lower median post/pretreatment ratio for migraine severity vs. placebo. Two studies found no significant difference between Mg and placebo in reducing pain intensity.
Veronese et al., Italy, 2020 [13]	Umbrella review	3 RCTs	186	28 to 46	i.v. Mg in acute migraine and oral Mg in chronic migraine	Strong evidence for decreased risk of frequency and intensity of migraine relapses in people with migraine was observed using the GRADE assessment.
Talandashti et al., Iran, 2024 [82]	SR and MA	3 RCTs	189 (frequency); 258 (severity); 150 (duration)	18 to 65	Oral Mg in migraine prophylaxis	Mg supplementation reduced migraine attacks, severity, and monthly migraine days vs. controls. However, notable heterogeneity was observed among the studies

* Studies reporting some significantly positive results are indicated in light blue. i.v.: intravenous; MA: meta-analysis; Mg: magnesium; MgSO_4_: magnesium sulfate; OR: odd ratio; RCT: randomized controlled trial; SR: systematic review.

In 2015, Teigen et al. [77] conducted a systematic review including four placebo-controlled trials assessing the efficacy of magnesium supplementation on migraine prevention [84,85,86,87]. Even if three of the four trials reviewed showed some benefit of magnesium supplementation on pain relief and migraine frequency when compared to placebo [84,85,87], the authors concluded that the strength of evidence was limited at that time. Conversely, a meta-analysis by Chiu et al. in 2016 [83] reviewed a broader sample of RCTs, including 11 studies on intravenous magnesium for acute migraines and 10 studies on oral magnesium for migraine prevention. Using odds ratios, they concluded that intravenous magnesium provided significant relief for acute migraines 15–45 min (OR = 0.23), 120 min (OR = 0.20), and 24 h (OR = 0.27) after magnesium administration. Similarly, oral magnesium reduced both the frequency (OR = 0.20) and the intensity (OR = 0.27) of migraine attacks [83]. Some of the trials included administered magnesium in combination with other substances, but nonetheless, this meta-analysis provides stronger evidence for the effectiveness of magnesium in both intravenous and oral forms for treating migraines and expanded upon the earlier analysis by including RCTs published in both English and Chinese, thereby broadening the scope, sample size, and external validity of the findings. Two other systematic reviews [78,79] reported benefits. The one by Von Lucker et al. [78] concluded that magnesium’s beneficial role in migraine prevention and its impact on quality of life are classified as Grade C evidence, indicating that it may be an effective treatment, based on the available data. This classification is supported by findings from five clinical trials (1990–2016) that showed a reduction in migraine days by 22–43% and indicated that high doses of oral magnesium daily (e.g., 600 mg of magnesium tricitrate) can be considered a safe and cost-effective addition in migraine care [78]. Another systematic review by Miller et al. [79] found potential benefits of intravenous magnesium sulfate in pain control beyond one hour, aura duration, and need for rescue analgesia. An extensive umbrella review of systematic reviews with meta-analyses of observational studies and RCTs aiming to map and grade all health outcomes associated with magnesium intake supplementation [13] found strong evidence for decreased risk of frequency and intensity of migraine relapses in people with migraine using the GRADE assessment. A recent systematic review and meta-analysis published in 2024 [82] including the effects of oral magnesium on migraine prophylaxis found a significant reduction in migraine attacks, severity, and monthly migraine days vs. controls. However, significant variability was observed across the studies. Conversely, a systematic review and meta-analysis among studies assessing the effectiveness of vitamins and minerals, including oral magnesium, on migraine prophylaxis [81] concluded that there was insufficient evidence of the effectiveness of oral magnesium for migraine prophylaxis in adults, and that robust, well-powered RCTs are necessary to thoroughly assess the effectiveness and safety of vitamins and minerals for preventing migraines. Another systematic review [80] including three RCTs found that one study observed a benefit, with oral magnesium showing a lower median post/pretreatment ratio for chronic migraine severity compared to a placebo. However, two other studies found no significant difference between magnesium and placebo in reducing pain intensity.

Overall, while recent evidence suggests that magnesium—both intravenous and oral—may be an effective treatment option, further RCTs with larger sample sizes and standardized designs are necessary to better understand magnesium’s efficacy and how it compares to current migraine pharmaceuticals.

### 4.4. Trials in Acute Migraine

Regarding the effectiveness of intravenous magnesium administration during acute migraine attacks, we found twelve RCTs, with favorable results in ten of these studies [74,88,89,90,91,92,93,94,95,96] and negative results in only two of them [97,98]. It should be noted that for this outcome, the results were more homogeneous due to the fact that the same magnesium salt preparation was used in all of the examined studies, i.e., magnesium sulphate in intravenous infusion. Table 2 provides a summary of the characteristics and results of the twelve RCTs assessing specifically the effectiveness of intravenous magnesium sulfate to improve pain and associated symptoms in patients with acute migraine episodes, in chronologic order.

Most studies showed that magnesium may have significant therapeutic potential for acute migraine relief. One of the first studies conducted by Mauskop et al. [74] found that 80% of the patients who received 1 g of intravenous magnesium were pain-free within 15 min of infusion (*p* < 0.001). Of the 20% who needed additional medications, the majority had chronic migraines (88%), and 37.5% of them had low levels of ionized magnesium (≤0.54 mmol/L) compared to 89% of patients who remained pain-free after 24 h. Most patients experienced mild flushing after the infusion [74]. Demirkaya et al. [88] compared 1 g of intravenous magnesium with placebo/normal saline and found that 86.6% of the magnesium group was pain-free within 30 min, compared to only 6.6% in the placebo group (*p* < 0.0001). Mild side effects like flushing and burning sensations on the face or neck were reported by 86.6% of the patients in both groups [88]. There were two studies performed by Bigal et al. [89,90] comparing 1 g of intravenous magnesium with placebo/normal saline. The first [90] found that magnesium was superior to placebo 30 and 60 min after the infusion, particularly in patients with aura. The second study [89] among few more patients observed that the patients with aura had better headache relief 1 h after magnesium administration (50% vs. 13.3%, *p* < 0.05), but there was no significant difference in relief for the patients without aura (33.3% with magnesium vs. 16.6% with placebo) [89]. Rahimdel et al. [91] compared the effects of magnesium sulfate and dihydroergotamine mesylate, considered a standard treatment, in the management of severe acute migraine headaches in 120 patients accessing the emergency department. The patients evaluated their pain on the visual analogue scale 30, 60, and 90 min after the intervention. The pain scores were significantly lower for patients receiving the magnesium infusion vs. those administered the placebo at 60 and 90 min, although not at 30 min, without serious side effects [91]. Abrishami et al. [92] performed a double-blind RCT among 30 patients admitted in the emergency ward fulfilling the international criteria for migraine diagnosis, receiving either intravenous normal saline or 2 g of magnesium sulfate. If, after 30 min, no headache relief was noted in both groups, in the second phase, magnesium sulfate and normal saline were administered in a crossover manner. The authors found that 93.3% of the patients receiving the magnesium infusion had a significant relief of their headache, and in all of them, there was complete relief of nausea, vomiting, photophobia, and phonophobia, with no patients receiving only saline having headache relief. In this group, the associated symptoms were relieved in two patients. In the second phase of the study, after magnesium administration to the placebo group, 93.3% of the patients had significant relief of both headache and associated symptoms. No side effects were observed, except flushing after magnesium infusion [92].

Sharami et al. [93], comparing the effects of magnesium sulfate with those of the combined use of dexamethasone and metoclopramide on relieving acute migraine headache among 70 patients (35 per arm) at an emergency department, found that magnesium sulfate was a more effective and fast-acting treatment compared to dexamethasone/metoclopramide [93]. A quasi-experimental study conducted at two hospitals by Baratloo et al. [94] compared 2 g of intravenous magnesium sulfate to 60 mg of caffeine citrate in patients with migraine. Both groups showed improvements in pain scores over the course of one hour, but the magnesium group experienced a significantly larger reduction in pain than the caffeine group (*p* < 0.001) [94]. Motamed et al. [95] examined the effect of magnesium sulfate, as an adjuvant treatment, in managing acute migraine in the emergency department, randomizing 40 patients who received 2 g of intravenous magnesium sulfate plus 10 mg of metoclopramide and 40 patients who received 10 mg of metoclopramide along with placebo. Although pain reduction was significant in both groups, the pain reduction slope for the group receiving the combination was significantly more severe, indicating that magnesium increased the effect of metoclopramide [95]. Finally, in the RCT conducted by Kandil et al. [96], patients were randomized to receive intravenous magnesium, prochlorperazine, or metoclopramide; a total of 157 patients were included, divided in three corresponding groups. There was a median decrease in the numbering rating scale of three points at 30 min across all three treatment arms. The median decrease in the pain rating scale at 30 or 60 min was similar for the three arms. There were no statistically significant differences in emergency department length of stay, rescue analgesia, or adverse effects. Prochlorperazine seemed to be more effective at 60 min but had greater adverse effects. The authors concluded that magnesium was non-inferior to metoclopramide and may serve as a safe alternative when agents such as prochlorperazine or metoclopramide are not appropriate [96].

There were two trials with non-favorable results. Cete et al. [98] compared 10 mg of intravenous metoclopramide, 2 g of intravenous magnesium sulphate, and normal saline. Pain reduction was similar among the groups (−38 for metoclopramide, −33 for magnesium, and −24 for placebo), but fewer patients in both the metoclopramide and the magnesium groups required rescue medications (38% and 44%, respectively) compared to the placebo group (65%) (*p* = 0.04). Dystonia was reported by 3% of the metoclopramide users, and 8% of the magnesium users experienced flushing. Thus, although the patients receiving the placebo required rescue medication more than the others, metoclopramide and magnesium had an analgesic effect similar to that of the placebo during the migraine attacks [98]. Corbo et al. [97] compared 2 g of intravenous magnesium with placebo/normal saline, with all patients receiving 20 mg of intravenous metoclopramide. Although pain reduction was similar for both groups, the patients in the metoclopramide plus placebo group showed a greater return to normal functioning on a 4-point disability scale (8% for the magnesium plus metoclopramide group vs. 17% for the metoclopramide plus placebo group, *p* < 0.05). The most common side effect was flushing (48% of the patients receiving magnesium vs. 22% of those receiving the placebo, which was not statistically significant) [97].

In summary, although the evidence comes from small studies, the effectiveness of intravenous magnesium sulfate in the management of acute migraine appears to be supported by the RTCs reviewed. Nevertheless, additional RCTs with larger sample sizes and standardized methodologies are needed to confirm the currently available favorable results.

**Table 2:** Summary of the characteristics and results of studies assessing the effectiveness of intravenous magnesium sulfate to improve pain and associated symptoms in patients with acute migraine episodes *.

Authors Country Year	Type of Study	No. of Participants	Age in Years (Mean)	Intervention	Assessment	Summary of Results
Mauskop et al., USA, 1995 [74]	Case-control	40	23 to 58 (40)	1 g i.v. MgSO_4_	Blood drawn for SiMg before i.v. infusion. Pain assessed with verbal scale (1–10)	Pain reduction of 50% after 15 min of infusion in 87.5% of patients. In 86%, complete relief or improvement persisted for ≥24 h. Pain relief lasted ≥24 h in 85.7% of patients with SiMg < 0.54 mmol/L, and in 16% with SiMg ≥ 0.54 mmol/L (*p* < 0.001) (OR = 27.9, *p* < 0.0001, 95% CI 5.4–194.4). Conclusion: SiMg may be useful in identifying migraine responsiveness to i.v. MgSO_4_.
Demirkaya et al., Turkey, 2001 [88]	RCT	30 (15 intervention– 15 placebo)	20 to 57 (35 ± 9)	1 g i.v. MgSO_4_ or 10 mL i.v. 0.9% saline	0 = No pain; 1 = mild pain, normal ADL; 2 = moderate pain, partially affecting ADL; 3 = severe pain, hindering ADL. Assessment of nausea, vomiting, photophobia, phonophobia, intolerance to exercise, irritability, and difficulty in concentrating. Assessment immediately after treatment, 30 min and 2 h later	Pain disappeared in 86.6% of the patients, diminished in 13.4%; accompanying symptoms disappeared in all. In the placebo group, decreased pain severity but persisting nausea, irritability, and photophobia in 6.6%, while other symptoms disappeared in 20% of the patients 30 min after placebo administration. All patients initially receiving the placebo and subsequently receiving MgSO_4_ responded as follows: in 93.3% of them, the attack ended; in 6.6%, pain intensity decreased; in all (100%), other symptoms disappeared; 86.6% had mild side effects that did lead to discontinuing MgSO_4_ administration. Conclusion: in view of these results, the effect of MgSO_4_ in acute migraine should be examined in large-scale studies.
Corbo et al., USA, 2001 [97]	RCT	44 (23 metoclopramide plus placebo; 21 metoclopramide plus MgSO_4_)	metoclopramide plus placebo 37 ± 8; metoclopramide plus MgSO_4_ 39 ± 12	20 mg of metoclopramide plus 0.9% saline or 20 mg of metoclopramide plus 2 g of MgSO_4_	VAS scores at 0, 15, 30, and 45 min	All had >50 mm improvement in pain VAS score, with smaller improvement in the MgSO_4_ group. The NNH in the MgSO_4_ plus metoclopramide group vs. metoclopramide alone group was 4 patients (95% CI 2 to 36). Conclusion: the addition of MgSO_4_ to metoclopramide may attenuate the effectiveness of metoclopramide, which may be explained by cerebral vasodilatation caused by MgSO_4_, although unproven. Since mainly women were in the trial, these data may not be generalizable to men.
Bigal et al., Brazil, 2002 [90]	RCT	42 (21 intervention– 21 placebo)	MgSO_4_ 27; placebo 28	1 g i.v. MgSO_4_ or 10 mL i.v. 0.9% saline	VAS scores 30 and 60 min after treatment	MgSO_4_ was superior to placebo (*p* < 0.05) 30 and 60 min after its administration; i.v. MgSO_4_ treatment was particularly effective in reducing aura compared to placebo.
Bigal et al., Brazil, 2002 [89]	RCT	60 (30 intervention–30 placebo)	MO: MgSO_4_ 31.4; placebo 27. MA: MgSO_4_ 27.1; placebo 28	1 g i.v. MgSO_4_ or 10 mL i.v. 0.9% saline	Seven parameters of analgesic evaluation and VAS to assess nausea, photophobia, and phonophobia	No difference between MgSO_4_ and placebo in pain relief and nausea in patients with migraine without aura but significant decreased intensity of photophobia and phonophobia. Significant pain relief and all associated symptoms in patients with migraine with aura associated with MgSO_4_ vs. placebo. Conclusion: MgSO_4_ can be used for the treatment of all symptoms in patients with migraine with aura or as an adjuvant therapy for associated symptoms in patients with migraine without aura.
Cete et al., Turkey, 2004 [98]	RCT	113 (37 metoclopramide; 36 magnesium; 40 placebo)	metoclopramide 40 ± 13; MgSO_4_ 40 ± 12; placebo 40 ± 11	10 mg i.v. metoclopramide or 2 g i.v. MgSO_4_ or 10 mL i.v. 0.9% saline	VAS scores at baseline and 30 min after treatment	All the groups had >25 mm VAS score improvement at 30 min without differences among them. The rescue medication requirement was higher in the placebo group. Recurrence rate in 24 h was also similar for both groups. Conclusion: although patients receiving placebo required more rescue medication, metoclopramide and MgSO_4_ had an analgesic effect similar to placebo in migraine attacks.
Rahimdel et al., Iran 2007 [91]	RCT	120 (60 MgSO_4_ –60 DHE)	>15	1 g i.v. MgSO_4_ or 1 mg i.v. DHE	VAS scores at 30, 60, and 90 min after treatment	VAS pain score was significantly lower with i.v. MgSO_4_ at 60 and 90 min, although not at 30 min, vs. DHE. Conclusion: treatment with 1 g of MgSO_4_ provided significant pain relief in migraine without serious side effects.
Abrishami et al., Iran 2010 [92]	RCT	30 (15 intervention–15 placebo)	NA	2 g i.v. MgSO_4_ or 10 mL i.v. 0.9% saline. If at 30 min no pain relief, MgSO_4_ and placebo crossover	VAS scores at baseline and 30 min, 2 h, and 24 h after treatment	Significant pain relief in 93.3% of patients receiving MgSO_4_. All had complete relief of nausea, vomiting, photophobia, and phonophobia. None in the placebo arm reported pain relief, but associated symptoms were relieved in 2 patients. In the second phase, after MgSO_4_ administration to placebo group, 93.3% of patients had significant relief of both headache and associated symptoms. No significant side effect of MgSO_4_, except flushing. Conclusion: clear-cut efficacy of 2 g MgSO_4_ infusion in acute migraine, but large-scale study is recommended.
Shahrami et al., Iran, USA, 2015 [93]	RCT	70 (35 MgSO_4_–35 dexamethasone/metoclopramide)	MgSO_4_ 36 ± 13; dexamethasone/metoclopramide 38 ± 11	1 g i.v. MgSO_4_ or 8 mg dexamethasone + 10 mg metoclopramide	Pain score based on 11-point standard NRS at baseline and after 20 min, 1 h, and 2 h	MgSO_4_ i.v. treatment associated with decreased pain severity at three time intervals (5.2 ± 1.7, 2.3 ± 1.9, and 1.3 ± 0.66 points, respectively), which was significantly better vs. baseline and vs. that at corresponding time intervals in the dexamethasone/metoclopramide group. Conclusion: MgSO_4_ was more effective and fast-acting vs. a combination of dexamethasone and metoclopramide for the treatment of acute migraine headaches.
Baratloo, et al., Iran, Egypt, 2017 [94]	RCT	70 (35 MgSO_4_–35 caffeine citrate)	MgSO_4_ 36 ± 2; caffeine citrate 30.2 ± 2	2 g i.v. MgSO_4_ or 60 mg caffeine citrate	VAS scores at baseline and 1 and 2 h after treatment	Both i.v. caffeine citrate and i.v. MgSO_4_ reduced pain scores significantly, but the MgSO_4_ group showed greater improvement than the caffeine citrate group after one hour (*p* < 0.001) and after two hours (*p* < 0.001). Conclusion: i.v. MgSO_4_ was more potent than caffeine citrate for the short-term management of migraine headache in ED.
Motamed et al., Iran, 2020 [95]	RCT	Group A: *n* = 40 Group B: *n* = 40	Group A: 20–30: 5 (12.5%) 31–40: 21 (52.5%) >40: 14 (35%) Group B: 20–30: 9 (22.5%) 31–40: 17 (42.5%) >40: 14 (35%)	Group A: 2 g MgSO_4_ plus 10 mg metoclopramide Group B: 10 mg metoclopramide plus 0.9% saline	NRS at baseline, 15, 30, and 45 min after intervention	Pain reduction was significant in both groups, but the reduction slope was more pronounced in group A. Conclusion: the use of MgSO_4_ along with metoclopramide increased the effect of metoclopramide in managing acute migraine. The findings could not support the independent effect of MgSO_4_ on the reduction of migraine headaches.
Kandil et al., USA, 2021 [96]	RCT	157 (61 MgSO_4_ –52 prochlorperazine–44 metoclopramide)	median MgSO_4_ 34; prochlorperazine 38; metoclopramide 38	2 g i.v. MgSO_4_, or 10 mg metoclopramide, or 10 mg prochlorperazine	Pain score based on an 11-point standard NRS at baseline and after 20 min, 1 h, and 2 h	No significant difference in NRS at 30 min between MgSO_4_, metoclopramide, and prochlorperazine. Prochlorperazine was more effective at 60 min but had greater adverse effects. No significant differences in ED LOS, rescue analgesia, or adverse effects. Conclusion: MgSO_4_ may be used as an adjunctive agent for the treatment of migraines or may serve as a safe alternative when agents such as prochlorperazine or metoclopramide are not appropriate.

* Studies reporting some significantly positive results are indicated in light blue. ADL: activities of daily living; CI: confidence interval; DHE: dihydroergotamine mesylate; ED: emergency department; i.v.: intravenous; LOS: length of stay; MA: migraine with aura; MgSO_4_: magnesium sulfate; MO: migraine without aura; NA: not available; NNH: number needed to harm; NRS: numbering rating scale; OR: odd ratio; RCT: randomized controlled trial; SiMg: serum ionized magnesium; VAS: visual analogue scale.

### 4.5. Trials in Chronic Migraine

Regarding the effect of oral magnesium supplementation in the prevention of chronic migraine, there is great heterogeneity in the magnesium salts used. We found eight RCTs, with seven of them reporting some favorable results [84,85,87,99,100,101,102], and only one reporting negative results [86]. Table 3 summarizes the main characteristics and results of the clinical trials assessing the effectiveness of magnesium supplementation in the prevention of chronic migraine.

Magnesium supplementation formerly demonstrated therapeutic efficacy in migraine patients, as evidenced by two double-blind, placebo-controlled randomized trials [84,85]. The first study involved 20 women with menstrual migraines [84]. These women were given two cycles of 360 mg of magnesium pyrrolidone carboxylic acid or a placebo daily, starting from ovulation until the first day of their period. Those receiving the active treatment experienced a notable reduction in both the frequency of headaches and the overall pain index. Interestingly, the pain total index was inversely correlated with the level of polymorphonucleated intracellular magnesium [84]. In a larger double-blind, RCT involving 81 adult patients with migraine (as per the International Headache Society criteria), the patients receiving active therapy also showed significant improvements [85]. The active treatment group, which received 600 mg of trimagnesium dicitrate in a water-soluble granular powder every morning, experienced a significant reduction (*p* < 0.05) in the frequency of the attacks (41.6%) compared to the placebo group (15.8%). Mild adverse events were diarrhea (18.6%) and gastric irritation (4.7%) [85].

A subsequent RCT by Wang et al. [102] involving 118 children aged 3 to 17 years, who were given either 9 mg/kg of daily oral magnesium oxide or a placebo, found that the treatment significantly reduced the number of headache days [102]. Koseoglu et al. [87] assessed the prophylactic effects of 600 mg of daily oral magnesium citrate supplementation in 30 migraine patients without aura, comparing the results with those of 10 patients receiving a placebo. After the magnesium treatment, there was a notable decrease in migraine attack frequency, severity, and P1 amplitude (measured in a visual evoked potential examination) compared to both pre-treatment values and the values of the placebo group [87]. In another RCT by Tarighat et al. [99], 133 patients with migraine were randomly assigned to three intervention groups: a magnesium oxide (500 mg/day) group, an L-carnitine (500 mg/day) group, and a group receiving a combination of magnesium plus L-carnitine (500 mg/day of each). A control group was also included. After 12 weeks of supplementation, significant reductions in migraine indicators, such as daily and monthly frequency and headache severity, were observed in all treatment groups (*p* < 0.05). The authors concluded that the three treatments, in addition to routine management, were effective for migraine prevention and recommended larger trials to further confirm these initial findings [99].

In a double-blind, crossover RCT, Karimi et al. [100] administered 63 patients 500 mg of magnesium oxide daily, followed by 800 mg of valproate sodium (400 mg every 12 h), or vice versa, for a duration of 24 weeks. The results revealed that the frequency and average duration of migraine attacks were similar in both groups, suggesting that magnesium oxide is as effective as valproate in preventing migraines without causing significant adverse effects [100]. Khani et al. [101] evaluated the effectiveness of concurrent magnesium and sodium valproate therapy for migraine prophylaxis, comparing it with magnesium plus placebo or sodium valproate plus placebo. They found a significant reduction in all migraine characteristics across all groups compared to baseline, with no statistically significant differences between the groups (*p* < 0.001). In the intragroup analysis, there was no significant difference in headache frequency between the group receiving sodium valproate plus placebo and the group receiving both magnesium and sodium valproate, in both the first and the third months. However, in the second month, the combined therapy group showed a significant reduction in frequency compared to the sodium valproate plus placebo group (*p* = 0.029). These findings suggest that magnesium may enhance the antimigraine effects of sodium valproate in combination therapy, potentially reducing the required sodium valproate dose for migraine prophylaxis [101].

In one trial involving 69 patients who took 242 mg of magnesium–u-aspartate hydrochloride trihydrate daily, no effect on migraines was observed [86]. Nearly half of the 35 patients who received magnesium experienced diarrhea, compared to a quarter of the 34 patients on a placebo, suggesting that the uncommon magnesium salt used might be poorly absorbed, which could help explain the lack of efficacy [86].

Overall, the existing evidence confirms that oral magnesium can be a useful measure to help in the prevention of migraine attacks. However, the available trials are small and of short duration, which is why additional studies are needed to determine the optimal magnesium supplementation formulation and dosage for migraine prophylaxis.

**Table 3:** Summary of the characteristics and results of clinical trials assessing the effectiveness of magnesium supplementation in the prevention of chronic migraine *.

Authors Country Year	Type of Study	No. of Participants	Age in Years (Mean)	Intervention	Assessment	Summary of Results
Facchinetti et al., Italy, 1991 [84]	RCT	35 (20 with menstrual migraine, intervention–15 without any history of migraine or PMS, placebo)	28 to 36 (Mg 30; placebo 28)	360 mg/day oral Mg pyrrolidone carboxylic acid or placebo divided t.i.d. for 4 months	PTI, MDQ during the 2 run-in cycles, and at months 2 and 4 of treatment	PTI decreased with both treatments at month 2; the Mg group showed the lowest values (*p* < 0.03). The *n* of days with headache and MDQ significantly decreased only in the Mg group. PTI and MDQ score decreased also at month 4 when Mg was supplemented, for all the patients. PTI was inversely correlated with PMN intracellular Mg++. Conclusion: Mg supplementation is useful for menstrual migraine prophylaxis and could be related to Mg deficiency.
Peikert et al., Germany, 1996 [85]	RCT	81 (43 intervention–38 placebo)	18–65 (Mg 44 ± 11; placebo 48 ± 10)	600 mg/day oral triMg dicitrate or placebo for 12 weeks	Headache diary recording headache *n*, intensity (VAS), and duration, dose of acute medication(s), and adverse events. Recorded data were checked and recorded by researchers every 4 weeks	Mg reduced attack frequency (−41.6%) vs. placebo (−15.8%) in week 9–12 compared to baseline (*p* < 0.05). The *n* of days with migraine and acute drug consumption also decreased significantly in the Mg group vs. placebo. Adverse events were diarrhea (18.6%) and gastric irritation (4.7%). Conclusion: high-dose Mg appears to be effective in migraine prophylaxis.
Pfaffenrath et al., Austria, Germany, Switzerland, 1996 [86]	RCT	69 (35 intervention–34 placebo)	18–64 (Mg 41 ± 12; placebo 40 ± 13)	486 mg/day oral Mg–u-aspartate hydrochloride trihydrate or placebo for 12 weeks	100 mm VAS	The *n* of responders (≥50% reduced intensity and duration of HA) was 1 in each group (Mg: 28.6%; placebo: 29.4%). No benefit regarding the *n* of migraine days or HA without center-specific differences. Assessments of treatment efficacy by doctor and patient were largely equivocal; 45.7% of patients under Mg reported mild adverse events (soft stools and diarrhea) vs. 23.5% under placebo.
Wang et al., USA, 2003 [102]	RCT	86 (42 intervention–44 placebo)	3–17 (Mg 41 ± 12; placebo 40 ± 13)	9 mg/kg/day oral Mg oxide divided t.i.d. or placebo for 16 weeks	Questionnaires completed by parents/guardians caring for younger children and by older participants themselves. Daily notes on a 16-week HA calendar: severity (6-point Wong–Baker FPRS), duration, and other symptoms (anorexia, photophobia, or phonophobia).	Significant decrease in HA frequency in the Mg group (*p* = 0.0037) vs. placebo group (*p* = 0.086), although the slopes were not significantly different. Mg-treated group had significantly lower headache severity (*p* = 0.0029) vs. placebo group. Conclusion: This study did not unequivocally determine whether oral Mg oxide was superior to placebo in preventing migraine headache in children but showed that it did lead to a significant reduction in headache days. Larger trials involving this safe, appealing complementary therapy are needed.
Köseoglu et al., Turkey, 2008 [87]	RCT	40 migraine patients without aura (30 intervention–10 placebo); 20 healthy persons as controls for VEP	20–55 (Mg 37 ± 9; placebo median 44; controls 35 ± 11)	600 mg/day oral Mg citrate or placebo daily for 3 months	VAS, VEP, brain SPECT imaging	HA frequency, severity, and P1 amplitude in VEP decreased after Mg vs. baseline (*p* < 0.001). Post/pretreatment ratios of HA frequency, severity, and P1 amplitude were significantly lower in Mg-treated group vs. placebo group. Cortical blood flow in inferolateral frontal, inferolateral temporal, and insular regions increased significantly after Mg treatment, while no significant changes with placebo. Conclusion: Mg is a beneficial agent in the prophylaxis of migraine without aura and might work with both vascular and neurogenic mechanisms.
Tarighat Esfanjani et al., Iran, 2012 [99]	RCT	133 (33 Mg; 35 L-carnitine; 30 Mg plus L-carnitine; 35 controls)	18–55 (Mg 37 ± 9; placebo median 44; controls 35 ± 11)	500 mg/day Mg oxide, or 500 mg/day L-carnitine, or Mg plus L-carnitine, or healthy controls for 12 weeks	checklist of HA/month, migraine days/month, and HA severity (0 = none; 1 = mild; 2 = moderate—activity impaired; 3 = severe—unable to function). MI calculated by multiplying the *n* of migraine days/month by migraine severity	Significant reduction in all migraine indicators in the 3 groups after 12 weeks. Conclusion: oral Mg oxide supplementation, L-carnitine, and their combination, besides routine treatments, could be effective in migraine prophylaxis; however, larger trials are needed to confirm these preliminary findings.
Karimi et al., Iran, 2021 [100]	RCT	63 (31 Mg; 32 NaV)	18–65 (Mg to NaV 36 ± 8; NaV to Mg 37 ± 9)	500 mg/day Mg oxide or 400 mg NaV b.i.d. for 8 weeks; 4-week washout period and crossover trial for 8 weeks	MIDAS scale; HIT-6 scores at baseline and at the end of each treatment phase	Significant decrease in the *n* of HA days/month, HA duration, and intensity after both treatments; no significant difference between Mg oxide and NaV. Conclusion: 500 mg Mg oxide appears to be as effective in migraine prophylaxis as NaV, without significant adverse effect.
Khani et al., Iran, 2021 [101]	RCT	Group A: *n* = 82 Group B: *n* = 70 Group C: *n* = 70	Group A: 35 ± 8 Group B: 37 ± 7 Group C: 34 ± 6	Group A: 200 mg NaV b.i.d. and placebo b.i.d. Group B: 200 mg NaV b.i.d. and 250 mg Mg oxide b.i.d. Group C: 250 mg Mg oxide b.i.d. and placebo b.i.d. All for 12 weeks.	MIDAS scale; HIT-6 scores at baseline and after 3 months treatment	Significant reduction in MIDAS and HIT-6 in all groups vs. baseline (*p* < 0.001). No statistically significant difference in HA frequency between groups A and B in the third month; three other parameters showed a significant reduction in group B vs. group A in the third month (*p* < 0.05). Group C was not different from groups A and B after 3 months (*p* < 0.001). Conclusion: Mg could enhance the antimigraine properties of NaV in combination therapy and reduce the NaV dose required for migraine prophylaxis.

* Studies reporting some significantly positive results are indicated in light blue. FPRS: face pain rating scale; HA: headache attack; HIT-6: headache impact test-6; MDQ: menstrual distress questionnaire; Mg: magnesium; MI: migraine index; MIDAS: migraine disability assessment; *n*: number; NaV: sodium valproate; PMN: polymorphonucleated cells; PMS: premenstrual syndrome; PTI: pain total index; RCT: randomized controlled trial; triMg: combination of 3 forms of magnesium; VAS: visual analogue scale; VEP: visual evoked potential.

## 5. Bioavailability of Different Magnesium Salts

It is notable to consider that different magnesium salts may have different characteristics. Indeed, elemental magnesium binds to organic and inorganic molecules to form compounds with varying behaviors. Despite differences in methodology, the studies on magnesium salts show that organic magnesium salts, like magnesium citrate, have better solubility and are absorbed more effectively than inorganic ones, such as magnesium oxide [103,104,105,106]. In fact, organic complexes are more soluble and less pH-sensitive than inorganic ones [106]. Inorganic salts include magnesium oxide, carbonate, and chloride, and organic salts include magnesium citrate, lactate, aspartate, and glycinate. Magnesium is mainly absorbed in the small intestine, especially in the distal jejunum and ileum, with minimal transcellular absorption in the colon. Absorption begins about 1 h after ingestion, reaching 80% after 6 h. Magnesium chelated with amino acids, like magnesium glycinate, is absorbed via the dipeptide transporter pathway [107]. Absorption is influenced by factors such as magnesium levels, age, dose, food, magnesium complex type, and formulation. Higher doses [106] and an empty stomach [108] increase absorption.

Clearly, intravenous magnesium has been shown to be more effective in rapidly (within 24 h) increasing serum magnesium concentrations from the baseline levels [109]. This is the reason why this route of administration was used in the clinical trials discussed above in situations of severe acute migraine with access to the emergency department. As shown by Reed et al. [109], intravenous magnesium administration is also indicated for the management of life-threatening ventricular arrhythmias or for preventing them in high-risk patients. The typical doses of intravenous magnesium used to correct hypomagnesemia during an acute episode are unlikely to be sufficient to address chronic hypomagnesemia, where a long-lasting use is needed in order to adequately replenish the total magnesium stores. It is important to note that very high doses of intravenous magnesium are administered in cases of pre-eclampsia and eclampsia, and the side effects are minimal. This is particularly significant, given that pregnant women require special care due to the potential risks for both the mother and the newborn, which confirms the safety of this magnesium treatment [110]. Nevertheless, it is crucial to note that magnesium supplementation can lead to diarrhea when taken in high doses; so, proper dose titration is essential. Additionally, evaluating renal function prior to supplementation is important, as magnesium supplementation should be used cautiously in patients with renal failure.

## 6. Mechanisms to Explain the Effect of Magnesium on Migraine

**Figure 6:**
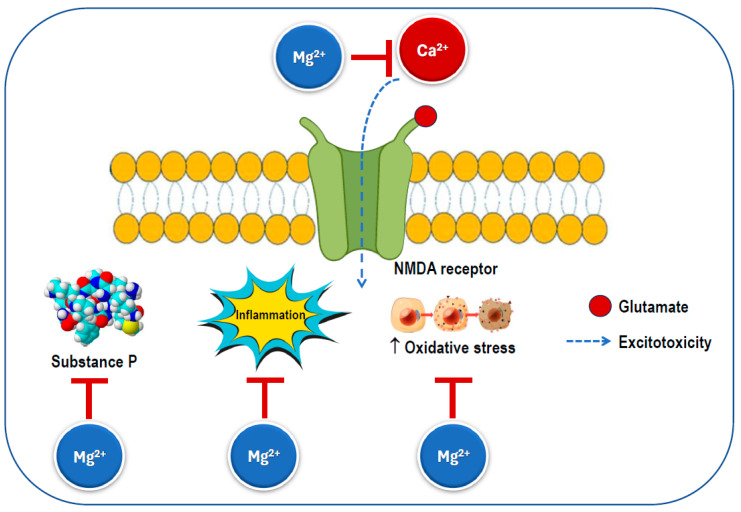
Possible mechanisms for the beneficial effects of magnesium on migraine. An excess of calcium influx through the NMDA receptor increases excitotoxicity, which triggers substance P release, inflammation, and oxidative stress. The magnesium ion blocks the ion channel of the NMDA receptor inhibiting calcium influx and preventing its excessive activation. NMDA: N-methyl-D-aspartate.

Several mechanisms have been proposed to explain the effects of magnesium on migraine discussed above (Figure 6). Magnesium has the ability to influence various neurochemical processes linked to the pathophysiology of migraine. While the precise mechanisms of migraine remain unclear, changes in central nervous system excitability, spontaneous neuronal depolarization, and dysfunctional mitochondrial activity have been associated with the condition. Given that glutamate is the primary excitatory neurotransmitter, it is frequently involved in discussions regarding the etiology, prevention, and treatment of migraine [15].

Magnesium can serve as a calcium channel blocker in neurons, where it is thought to prevent the overactivation of excitatory synapses, such as those involving the NMDA receptor, which is known to play a significant role in pain transmission and cortical spreading depression [111]. Additionally, it has been shown to reduce inflammation by inhibiting pro-inflammatory intracellular signaling, including the nuclear factor kappa B pathway [11,112]. Notably, disruptions in the magnesium balance within the brain have been observed in several neurological disorders [54]. Magnesium is also an essential factor in mitochondrial function and helps reduce membrane permeability, thereby decreasing the likelihood of spontaneous neuronal depression caused by hyperexcitability [113]. Magnesium helps maintain the calcium balance by interacting with NMDA glutamate receptors, regulates the release of substance P, and controls nitric oxide production. Ionized magnesium also impacts the vascular tone and can inhibit cortical spreading depression in animal models [114].

Magnesium deficiency has been connected to cortical spreading depression (CSD), which is believed to play a role in the aura phase of migraines [115,116], as well as to imbalances in neurotransmitter release and platelet activity [115]. In CSD, magnesium deficiency triggers the release of substance P, a neuropeptide that functions as both a neurotransmitter and a neuromodulator [117], which promotes a reversible decline in blood–brain barrier integrity [118]. This release may affect sensory fibers, potentially leading to headache pain. Spreading depression is specified by the breakdown of ion homeostasis related to a temporary cessation of neuronal function and is understood to play a role in migraine pathogenesis and need the release of glutamate. NMDA receptors play a critical role in the propagation of this process [116].

Magnesium has been found to reduce the levels of circulating CGRP, which plays a role in the development of migraines by causing the dilation of intracranial blood vessels and generating pain-related stimuli [115,119]. Magnesium from external sources may help alleviate different aspects of neurogenic inflammation due to its involvement in regulating NMDA glutamate receptors. These receptors are critical for pain transmission in the nervous system [111,120], cerebral blood flow regulation [121,122], and the initiation and spread of CSD. Studies have shown that ionized magnesium can inhibit CSD by modulating glutamatergic neurotransmission, blocking the NMDA receptor calcium channel, and influencing the signaling of the cyclic adenosine monophosphate response element-binding protein [22]. The regulation of cerebral blood flow by circulating nitric oxide plays a role in headaches and is known to be affected by magnesium intake [115,123]. Additionally, magnesium can promote vasodilation directly by blocking calcium-sensitive potassium channels on smooth muscle cells [122]. Serotonin, a powerful cerebral vasoconstrictor released from platelets during a migraine attack, may also play a significant role in migraine pathogenesis [124], while magnesium is a key modulator of the serotoninergic system [125].

## 7. Conclusions

Magnesium has been considered for over thirty years in the treatment of migraine, a common and disabling chronic condition. This ion has multiple effects on the nervous system, including those derived from its interaction with NMDA receptors, the inhibition of voltage-gated calcium channels, and a reduction in oxidative stress, inflammation, and the release of substance P. These mechanisms may contribute to the role of magnesium in controlling migraine. Magnesium is also a supplement with relatively rare serious side effects and shows promise in addressing the progression of migraine symptoms. While the current data support the benefits of both intravenous and oral magnesium administration for the management of acute migraine episodes requiring immediate treatment as well as in the prevention of migraine, larger RCTs are needed to further validate the effectiveness of magnesium as a treatment option. Although these trials are needed, considering the evidence from the last decades, magnesium could be included in guidelines and protocols for the treatment of both acute and chronic migraine, as indicated by the studies included in this review. Magnesium supplementation could be considered as an add-on therapy when other treatments are minimally effective. An initial therapeutic trial with magnesium, followed, if necessary, by other established and new treatments is also possible.

Thus, oral magnesium supplements are a cost-effective and well-tolerated option for treating migraine patients, alone or in combination with other compounds, which may indeed help decrease the frequency of attacks, lower treatment costs, and minimize the risk of severe side effects.
